# Evaluating the Association of Serum 25-Hydroxyvitamin D, Osteocalcin, Periostin, and Estradiol Levels With Hip Fracture Type and Severity in Patients Attending a Tertiary Care Center

**DOI:** 10.7759/cureus.115558

**Published:** 2026-08-31

**Authors:** Yogesh Kadam, Navin Satyanaryan, Harish Bagewadi, Priyanka M, Renuka S Melkundi, Shivalee A, Rohini Kallur

**Affiliations:** 1 Orthopaedics, Gulbarga Institute of Medical Sciences, Kalaburagi, IND; 2 Biochemistry, Gulbarga Institute of Medical Sciences, Kalaburagi, IND; 3 Pharmacology, Gulbarga Institute of Medical Sciences, Kalaburagi, IND; 4 Anatomy, Gulbarga Institute of Medical Sciences, Kalaburagi, IND; 5 Otolaryngology, Gulbarga Institute of Medical Sciences, Kalaburagi, IND; 6 Research, Gulbarga Institute of Medical Sciences, Kalaburagi, IND

**Keywords:** 25-hydroxyvitamin d, estradiol, hip fracture, osteocalcin, periostin, singh index

## Abstract

Background and objective

25-Hydroxyvitamin D (25(OH)D) status and bone turnover markers are considered important determinants of bone quality and are associated with the risk of hip fractures. However, the exact relationship between 25(OH)D, bone turnover markers, and the type and severity of hip fractures remains unclear. Hence, this study aimed to investigate the relationship between serum vitamin D levels and selected bone turnover biomarkers among patients with hip fractures.

Methods

According to the inclusion and exclusion criteria, a total of 70 patients with hip fractures were enrolled in this prospective study conducted between July 2025 and June 2026. The hip fracture patients were classified into intracapsular (IC) and extracapsular (EC) fracture groups. Serum concentrations of 25(OH)D, osteocalcin, periostin, and estradiol (E2) were measured using commercially available enzyme-linked immunosorbent assay (ELISA) kits from Elabscience (Houston, TX).

Results

The serum levels of 25(OH)D and E2 were significantly lower in patients with EC fractures than in those with IC fractures. In contrast, osteocalcin and periostin levels were significantly higher in the EC group than in the IC group. A statistically significant positive correlation (p < 0.05) was observed between serum osteocalcin and 25(OH)D levels and Singh index grades I-II and III-IV. Conversely, a statistically significant negative correlation (p < 0.05) was observed between serum periostin and E2 levels and Singh index grades I-II and III-IV. However, no statistically significant correlation was observed for Singh index grades V-VI.

Conclusions

In both the EC and IC fracture groups, serum 25(OH)D levels were significantly associated with Singh index grades I-II and III-IV. Lower serum levels of 25(OH)D and E2 and higher levels of osteocalcin and periostin were observed in patients with EC fractures compared with those with IC fractures. Further studies are warranted to improve our understanding of the roles of vitamin D and other bone turnover markers in orthopedic healthcare.

## Introduction

Hip fractures are a significant health concern, particularly among the elderly population, and are among the most common reasons for orthopedic consultations. Hip fractures are associated with increased morbidity, mortality, and healthcare costs [1]. Elderly individuals, who are often confined indoors and are at increased risk of falls, are especially susceptible to vitamin D deficiency and its adverse effects on bone health. Vitamin D deficiency is also commonly observed in patients with chronic renal and hepatic diseases [2].

Vitamin D maintains calcium homeostasis through the binding of 1,25-dihydroxyvitamin D₃ (1,25(OH)₂D₃) to vitamin D receptors. However, the half-life of 1,25(OH)₂D₃, the biologically active form of vitamin D, is too short for it to be a reliable indicator of vitamin D status [3]. Osteocalcin is a small protein consisting of 49 amino acids and is encoded by the bone gamma-carboxyglutamate protein (BGLAP) gene. It is synthesized by osteoblasts, and its serum concentration is widely regarded as a biochemical marker of bone formation, reflecting both the number and activity of osteoblasts [4]. Periostin is predominantly expressed in the periosteum, which covers most bone surfaces and plays an important role in bone remodeling and metabolism [5]. Furthermore, lower serum levels of 17β-estradiol (E2) have been reported to be associated with an increased risk of fractures during the menopausal transition [6].

Despite increasing evidence regarding the role of vitamin D and bone turnover biomarkers in skeletal health, the relationship between serum 25(OH)D, osteocalcin, periostin, and E2 levels and the risk of hip fractures remains incompletely understood. A better understanding of these associations may facilitate the development of targeted strategies for fracture prevention and management beyond conventional orthopedic care. Therefore, the present study was undertaken to investigate the relationship between serum vitamin D levels and selected bone turnover biomarkers in patients with hip fractures.

## Materials and methods

Study design

This hospital-based prospective observational study was conducted at a single tertiary care center between July 2025 and June 2026.

Patient recruitment

Participants for this study were recruited from both the outpatient (OPD) and inpatient (IPD) units of the Department of Orthopedics. The research was conducted from July 2025 to June 2026. Prior to initiation, approval was obtained from the Institutional Ethics Committee (Approval No: GIMS/KLB/PHARMA/IEC/293/2024-25 dated 08-07-2024). Written informed consent was obtained from all participants before enrollment. All procedures adhered to institutional and ethical guidelines, and written informed consent was secured from each participant before enrolment.

Eligibility criteria

Inclusion Criteria

All patients aged ≥ 45 years of both genders who were diagnosed with hip fractures, including femoral neck and intertrochanteric fractures, following a trivial trauma, such as a slip and fall while standing or walking, were recruited for the study.

Exclusion Criteria

Patients with a significant history of trauma, such as road traffic accidents/fall from height and pathological fractures, metabolic-related diseases (including thyroid disease, renal insufficiency, hepatic insufficiency, malignancy), and patients taking vitamin D supplementation were excluded from the study.

Standard radiographic evaluation included anteroposterior radiographs of the pelvis with both hips and a lateral radiograph of the affected hip. Based on fracture location, participants were categorized into two groups: the intracapsular (IC) fracture group (n = 34) and the extracapsular (EC) fracture group (n = 36). Bone quality was assessed using the Singh Index, a radiographic classification system based on the visibility of trabecular patterns in the proximal femur [7].

Previous studies by Xu et al. [8] and Kalavakonda et al. [9] have shown good predictive values of the Singh Index for the risk of intertrochanteric fractures. A study by Liu Z et al. [10] has shown that the Singh Index has a significant correlation with the mechanical strength of the hip. Participants were further classified into three categories: Singh grade I-II, Singh grade III-IV, and Singh grade V-VI. Fracture type and trabecular architecture were independently assessed by two orthopedic surgeons and two radiologists to enhance measurement reliability.

We acknowledge the presence of intra- and inter-observer variation. The intra-observer reliability was relatively consistent when the same radiographs were re-evaluated over time using the exact Singh grades: I-II, III-IV, and V-VI. The intraclass correlation coefficient (ICC) ranged from 0.50 to 0.75, indicating moderate reliability. The inter-observer reliability showed moderate agreement when different clinicians attempted to assign exact scores using the six-grade Singh Index. For the graded assessment of proximal femoral trabecular patterns (Singh grades I-II, III-IV, and V-VI), the kappa values ranged from 0.41 to 0.60 (95% CI), indicating moderate agreement.

Sample size estimation

A total of 70 participants were included in the study. The minimum sample size was calculated using Epi Info software (Version 7.1, Centers for Disease Control and Prevention, USA), based on the methodology described by Khaled Fahim et al. [11]. The study's measurable parameters had a 6% margin of error, with a 7% prevalence rate of osteoporosis reported in a previous study by Kaushal et al. [12]. The calculation was based on a 95% confidence level, 80% statistical power, and a 6% margin of error, with an alpha (α) value of 0.05. The final calculated sample size was 70 participants.

Blood sample collection and biochemical analysis

Blood samples were collected at the Multidisciplinary Research Unit. Approximately 5 mL of venous blood was obtained from each participant under aseptic conditions. The samples were centrifuged at 3000 rpm for 10 minutes, and the separated serum was stored at 2-8 °C until analysis. The serum concentrations of specific biomarkers were determined using commercially available human ELISA kits according to the manufacturer’s instructions (Elabscience, Houston, TX). The biomarkers assessed included osteocalcin (Cat. No. E-EL-H1343; sensitivity: 0.75 ng/mL; detection range: 1.25-80 ng/mL); periostin (Cat. No. E-EL-H6160; sensitivity: 0.09 ng/mL; detection range: 0.16-10 ng/mL); 25(OH)D (Cat. No. E-EL-0015; sensitivity: 1.88 ng/mL; detection range: 3.13-200 ng/mL); and estradiol (E2) (Cat. No. ELK10157; sensitivity: 12.69 pg/mL; detection range: 46.88-3000 pg/mL). The intra- and inter-assay coefficients of variation were maintained below 10%.

Statistical analysis

Statistical analysis was performed using SPSS Statistics software version 19.0. Continuous variables were expressed as mean ± standard deviation (SD), while categorical variables were presented as frequencies and percentages. Comparisons between categorical variables were performed using the chi-square test. Differences in continuous variables between the IC and EC fracture groups were analyzed using the independent-samples Student's t-test. Comparisons among Singh Index categories were performed using one-way analysis of variance (ANOVA). Pearson's correlation coefficient was used to evaluate the association between serum biomarker levels and Singh Index grades. A p-value < 0.05 was considered statistically significant for all analyses.

## Results

Table 1 presents the demographic and clinical characteristics of the study participants. The serum SGPT and SGOT levels were higher in the EC group than in the IC group; however, the difference was not statistically significant. Furthermore, serum calcium and phosphorus levels were significantly lower in the EC group than in the IC group (p < 0.05).

**Table 1:** Comparison of sociodemographic profile and laboratory parameters ^*^P < 0.05 versus IC group Chi-square test and Student’s t-test were applied IC: intracapsular; EC: extracapsular; SD: standard deviation; SGPT: serum glutamic pyruvic transaminase; SGOT: serum glutamic-oxaloacetic transaminase

Parameters	IC group (n = 34)	EC group (n = 36)	Test value	P-value
Age, years, mean ± SD	68.7 ± 0.49	71.8 ± 1.28	t = 4.42	0.094
Male-to-female ratio	11:23	10:26	χ² = 0.17	0.676
BMI, kg/m², mean ± SD	22.9 ± 2.31	23.6 ± 1.85	t = 1.41	0.163
Serum calcium, mg/dL, mean ± SD	7.32 ± 2.6	6.08 ± 1.4^*^	t = 2.47	0.016
Serum phosphorus, mg/dL, mean ± SD	3.1 ± 2.4	1.8 ± 2.1^*^	t = 2.40	0.019
Serum creatinine, mg/dL, mean ± SD	0.8 ± 0.062	0.9 ± 0.053	t = 1.33	0.252
SGPT, U/L, mean ± SD	35.99 ± 3.9	38.99 ± 2.2	t =1.57	0.32
SGOT (U/L)	13.13±3.2	15.53 ± 2.8	t = 1.62	0.36

As shown in Table 2, serum 25(OH)D and estradiol levels were significantly lower in patients belonging to the EC group compared with those in the IC group. In contrast, serum osteocalcin and periostin levels were significantly higher in the EC group than in the IC group.

**Table 2:** Comparison of serum 25(OH)D, osteocalcin, periostin, and estradiol levels in hip fracture patients ^*^P < 0.05 versus IC group Student’s t-test was applied 25(OH)D: 25-hydroxyvitamin D; IC: intracapsular; EC: extracapsular; SD: standard deviation

Serum values	IC group (n = 34), mean ± SD	EC group (n = 36), mean ± SD	(t)-value	P-value
(25(OH)D), ng/mL	13.5 ± 4.4	8.1 ± 5.6^*^	4.49	0.032
Osteocalcin, ng/mL	30.4 ± 4.8	50.6 ± 3.5	20.12	0.017
Periostin, ng/mL	158.4 ± 3.5	186.5 ± 4.12	30.64	0.012
Estradiol, pg/mL	9.03 ± 2.3	7.24 ± 1.03^*^	4.17	0.043

As presented in Table 3, the serum levels of osteocalcin and periostin were higher in Singh (I - II) and Singh (III - IV) index patients, which was statistically significant (p < 0.05) when compared with Singh (V - VI) grade. The serum levels of calcium, phosphorus, 25(OH)D, and estradiol were lower in Singh (I - II) and Singh (III - IV) index patients, which was statistically significant (p < 0.05) when compared with Singh (V - VI) grade. The serum SGPT and SGOT levels were higher in Singh (I - II) and Singh (III - IV) index patients when compared with Singh (V - VI) grade. These differences were not statistically significant (p < 0.05).

**Table 3:** Comparison of serum calcium, 25(OH)D, osteocalcin, periostin, and estradiol levels in different grades of Singh index ^*^P < 0.05 versus Singh grade V–VI; ^†^p < 0.01 versus Singh grade V–VI; ^‡^p < 0.05 versus Singh grade III–IV One-way analysis of variance (ANOVA) was used for comparisons among the groups 25(OH)D: 25-hydroxyvitamin D; SD: standard deviation; SGPT: serum glutamic pyruvic transaminase; SGOT: serum glutamic-oxaloacetic transaminase

Serum values	Singh (V - VI) (n = 12), mean ± SD	Singh (III - IV) (n = 24), mean ± SD	Singh (I - II) (n = 34), mean ± SD	(F)-value	P-value
Calcium, mg/dL	13.08 ± 2.6	11.12 ± 2.1^*^	9.05 ± 1.4^*^	19.69	0.038
25(OH)D, ng/mL	14.2 ± 3.8	12.5 ± 5.8^*^	9.24 ± 2.2^*^	6.80	0.032
Phosphorus, mg/dL	3.2 ± 1.2	2.1 ± 0.8^*^	1.8 ± 0.6^*^	14.13	0.044
SGPT, U/L	32.73 ± 5.8	36.56 ± 3.6	38.92 ± 4.2	13.08	0.282
SGOT, U/L	13.31 ± 1.2	14.26 ± 2.5	15.73 ± 3.6	4.85	0.374
Osteocalcin, ng/mL	30.5 ± 3.4	38.2 ± 5.8^*^	44.6 ± 3.5^†^	38.92	0.002
Periostin, ng/mL	151.3 ± 2.35	166.2 ± 3.12^*^	182.7 ± 3.01^†, ^^‡^	131.73	0.002
Estradiol, pg/mL	9.56 ± 3.52	8.01 ± 3.13^*^	7.24 ± 4.01^*^	1.52	0.042

In both the IC and EC groups, a significant positive correlation was observed between serum 25(OH)D levels and Singh grades I - II and III-IV (p < 0.05). In contrast, a weak positive correlation was observed between serum 25(OH)D levels and Singh grade V - VI; however, this association was not statistically significant (p > 0.05) (Table 4).

**Table 4:** Correlation between serum 25(OH)D and Singh index in hip fracture patients ^*^Statistically significant Pearson’s correlation test was applied. (r) - value: Pearson's correlation coefficient 25(OH)D: 25-hydroxyvitamin D; IC: intracapsular; EC: extracapsular

Singh index	Serum 25(OH)D (r) - value in the IC group	P-value	Serum 25(OH)D (r) - value in the EC group	P-value
Singh (V - VI) (5.16)	0.16	0.064	0.19	0.075
Singh (III - IV) (3.76)	0.22	0.038^*^	0.27	0.033^*^
Singh (I - II) (1.92)	0.62	0.022^*^	0.58	0.026^*^

A significant positive correlation was observed between serum osteocalcin levels and Singh grades I - II and III - IV in both the IC and EC groups (p < 0.05). However, only a weak positive correlation was found between serum osteocalcin levels and Singh grade V-VI, which was not statistically significant (p > 0.05) (Table 5).

**Table 5:** Correlation between serum osteocalcin and Singh index in hip fracture patients *Statistically significant Pearson’s correlation test was applied. (r) - value: Pearson's correlation coefficient IC: intracapsular; EC: extracapsular

Singh index	Serum osteocalcin (r) - value in the IC group	P-value	Serum osteocalcin (r) - value in the EC group	P-value
Singh (V - VI) (5.16)	0.19	0.072	0.23	0.064
Singh (III - IV) (3.76)	0.23	0.043^*^	0.26	0.039^*^
Singh (I - II) (1.92)	0.51	0.032^*^	0.51	0.028^*^

Serum periostin levels showed a negative correlation with Singh index grades in both the IC and EC groups. The negative correlation was statistically significant for Singh grades III - IV and I - II in both groups (p < 0.05). In the EC group, a significant negative correlation was also observed for Singh grade V - VI, whereas the corresponding correlation in the IC group was not statistically significant (p > 0.05) (Table 6).

**Table 6:** Correlation between serum periostin and Singh index in hip fracture patients Pearson’s correlation test was applied. (r) - value: Pearson's correlation coefficient IC: intracapsular; EC: extracapsular

Singh index	Serum periostin (r) - value in the IC group	P-value	Serum periostin (r) - value in the EC group	P-value
Singh (V - VI) (5.16)	-0.24	0.21	-0.28	0.042
Singh (III - IV) (3.76)	-0.25	0.038	-0.32	0.036
Singh (I - II) (1.92)	-0.46	0.032	-0.57	0.028

As shown in Table 7, serum estradiol levels demonstrated a negative correlation with Singh index grades in both the IC and EC groups. Significant negative correlations were observed for Singh grades III - IV and I - II in both groups (p < 0.05). In the EC group, a significant negative correlation was also observed for Singh grades V - VI, whereas the corresponding correlation in the IC group was not statistically significant (p > 0.05).

**Table 7:** Correlation between serum estradiol and Singh index in hip fracture patients Pearson’s correlation test was applied. (r) - value: Pearson's correlation coefficient IC: intracapsular; EC: extracapsular

Singh index	Serum estradiol (r) - value in the IC group	P-value	Serum estradiol (r) - value in the EC group	P-value
Singh index (V - VI) (5.16)	-0.28	0.34	-0.32	0.035
Singh index (III - IV) (3.76)	-0.30	0.032	-0.36	0.034
Singh index (I - II) (1.92)	-0.32	0.022	-0.58	0.028

## Discussion

In our study, the findings demonstrated that fragility hip fractures occurred more frequently in women than in men. Serum 25(OH)D concentrations were markedly reduced in both IC and EC fracture groups, indicating a high prevalence of hypovitaminosis D among patients with hip fractures. The observed findings are in agreement with a previous study by Han et al. [13], which reported severe vitamin D deficiency in 83.2% of patients with hip fractures, while an additional 11.9% exhibited vitamin D insufficiency, leaving only a small proportion with normal vitamin D status. Similarly, Fatma et al. [14] documented a high frequency of suboptimal vitamin D levels among elderly individuals presenting with proximal femoral fractures. Long-term observational evidence also supports the role of vitamin D in fracture prevention. Buchebner et al. [15] demonstrated that persistent vitamin D insufficiency over a five-year period was associated with an increased 10-year risk of hip and major osteoporotic fractures in elderly women. Previous studies have demonstrated a significant relationship between the Singh index and bone mineral density, supporting its utility as a practical screening tool for osteoporosis and fracture risk assessment [16].

The present study demonstrated that patients with EC hip fractures had significantly lower serum calcium concentrations than those with IC fractures. Similarly, Li et al. [17] observed significantly reduced serum calcium levels among patients with intertrochanteric fractures compared with those sustaining femoral neck fractures. Reduced calcium levels in elderly individuals may be attributable to inadequate dietary intake, age-related nutritional deficiencies, and the presence of chronic medical conditions such as hypertension and diabetes mellitus, all of which may contribute to compromised nutritional status and skeletal health [18]. Consequently, reduced serum calcium levels may be closely linked to underlying vitamin D deficiency [19]. In the present study, the correlation analysis revealed a significant positive association between serum 25(OH)D concentrations and Singh Index grades III-IV and I-II in both IC and EC fracture groups (p < 0.05). Although dual-energy X-ray absorptiometry (DEXA) provides valuable information regarding bone density, its ability to predict individual fracture risk is not absolute, and repeated measurements may be required to monitor treatment response [20].

In the present study, the correlation analysis further showed a significant positive relationship between serum osteocalcin levels and lower Singh index grades (I-II and III-IV) in both IC and EC fracture groups (p < 0.05). Our study findings align with a previous study by Ghalenavi et al. [21]. The observed increase in osteocalcin concentrations among patients with more severe osteoporosis may reflect enhanced osteoblastic activity occurring as a compensatory response to accelerated bone remodeling and skeletal deterioration. In this study, the correlation analysis further showed a significant association between serum periostin levels and lower Singh index grades (I-II and III-IV) within the EC fracture group (p < 0.05). In contrast, only a weak association was observed between periostin levels and Singh grades V-VI, which did not reach statistical significance. Our findings are in accordance with a previous study by Guo et al., in which an increased risk of fracture was found in patients with high serum periostin levels [22].

Similarly, Farrokhi et al. [23] reported increased circulating periostin concentrations in patients with acute hip fractures and proposed that periostin may contribute to the early stages of fracture healing and bone regeneration. Serum E2 levels were significantly lower in patients with EC fractures compared with those with IC fractures. Analysis according to Singh index categories further revealed significantly lower E2 levels among patients classified within Singh grades I-II and III-IV compared with those in Singh grades V-VI.

In the present study, the correlation analysis demonstrated a significant negative association between serum E2 levels and Singh grades I-II and III-IV in both IC and EC fracture groups (p < 0.05). Although a negative correlation was also observed in patients with Singh grades V-VI, the association did not reach statistical significance. This finding may be explained by the timing of blood sample collection and a biphasic response, in which the periostin level fell in the first 48 hours of injury and then progressively increased over the following weeks as the bone repaired. Our findings are supported by previous studies; Nethander et al. [24] and Sharifi et al. [25] provided evidence for a causal relationship between E2 and fracture risk, emphasizing the protective role of estrogen in bone metabolism. Similarly, Kantharaj et al. [26] reported that elderly men with osteoporosis of the femoral neck were more likely to exhibit E2 deficiency, further supporting the contribution of low estrogen levels to skeletal fragility.

Strengths and limitations

The strengths of this study are that it adds to the existing evidence regarding the relationship between serum calcium, 25(OH)D, osteocalcin, periostin, and E2 levels in patients with different types of hip fractures and varying degrees of osteoporosis as assessed by the Singh index. By evaluating both IC and EC fracture patterns and including participants of both sexes, the study provides a broader perspective on the potential role of bone turnover markers in fracture risk assessment and bone health evaluation.

However, this study has certain limitations. Firstly, the study was conducted at a single tertiary care center with a relatively small sample size, which may limit the generalizability of the findings to other populations. Secondly, serum biomarker and hormone levels were measured at a single time point following fracture presentation, and therefore temporal variations could not be assessed. Thirdly, bone mineral density was evaluated using the Singh index rather than DEXA, which remains the gold standard for osteoporosis assessment. In addition, the study did not include longitudinal follow-up to determine the impact of these biomarkers on fracture healing, functional recovery, or future fracture risk. The absence of interventional evaluation, such as vitamin D or hormonal supplementation, further limits the ability to establish causal relationships.

## Conclusions

The findings of the present study highlight the potential clinical relevance of bone turnover biomarkers in assessing bone quality and fracture risk. Significant associations were observed between serum calcium, 25(OH)D, osteocalcin, periostin, and estradiol levels and Singh index grades, suggesting their possible role in reflecting skeletal health and osteoporosis severity. Future investigations should involve larger multicenter cohorts and prospective longitudinal designs to validate these observations. Randomized controlled trials evaluating the effects of vitamin D supplementation and the modulation of bone turnover markers on fracture prevention and healing outcomes would provide valuable clinical evidence. A better understanding of the interactions among vitamin D, osteocalcin, periostin, estradiol, and skeletal metabolism may contribute to the development of more effective strategies for osteoporosis management and the reduction of the hip fracture burden in older adults.

## References

[1] Veronese N, Maggi S (2018). Epidemiology and social costs of hip fracture. Injury.

[2] Parizadeh SM, Rezayi M, Jafarzadeh-Esfehani R (2021). Association of vitamin D status with liver and kidney disease: a systematic review of clinical trials, and cross-sectional and cohort studies. Int J Vitam Nutr Res.

[3] Torbergsen AC, Watne LO, Wyller TB, Frihagen F, Strømsøe K, Bøhmer T, Mowe M (2015). Vitamin K1 and 25(OH)D are independently and synergistically associated with a risk for hip fracture in an elderly population: a case control study. Clin Nutr.

[4] Diaz-Franco MC, Franco-Diaz de Leon R, Villafan-Bernal JR (2019). Osteocalcin‑GPRC6A: an update of its clinical and biological multi‑organic interactions (review). Mol Med Rep.

[5] Li C, Li X, Wang X (2020). Periostin mediates oestrogen-induced osteogenic differentiation of bone marrow stromal cells in ovariectomised rats. Biomed Res Int.

[6] Cauley JA, Ruppert K, Lian Y (2019). Serum sex hormones and the risk of fracture across the menopausal transition: study of women's health across the nation. J Clin Endocrinol Metab.

[7] Singh M, Nagrath AR, Maini PS (1970). Changes in trabecular pattern of the upper end of the femur as an index of osteoporosis. J Bone Joint Surg Am.

[8] Xu Z, Tian G, Liu C, Xie Y, Zhang R (2022). The predictive value of the Singh index for the risk of InterTAN intramedullary fixation failure in elderly patients with intertrochanteric fractures. BMC Musculoskelet Disord.

[9] Kalavakonda V, Mohamed S, Abhay L, Muthu S (2024). Automated screening of hip X-rays for osteoporosis by Singh's index using machine learning algorithms. Indian J Orthop.

[10] Liu Z, Gao H, Bai X, Zhao L, Li Y, Wang B (2017). Evaluation of Singh index and Osteoporosis Self-Assessment Tool for Asians as risk assessment tools of hip fracture in patients with type 2 diabetes mellitus. J Orthop Surg Res.

[11] Khaled Fahim N, Negida A (2018). Sample size calculation guide - part 1: how to calculate the sample size based on the prevalence rate. Adv J Emerg Med.

[12] Kaushal N, Vohora D, Jalali RK, Jha S (2018). Prevalence of osteoporosis and osteopenia in an apparently healthy Indian population - a cross-sectional retrospective study. Osteoporos Sarcopenia.

[13] Han J, Cho Y, Jee S, Jo S (2020). Vitamin D levels in patients with low-energy hip fractures. Hip Pelvis.

[14] Fatma H, Makhloufi-Dahou C, Nour El Houda K (2024). AB0311 vitamin D status and proximal femur fracture. Ann Rheum Dis.

[15] Buchebner D, McGuigan F, Gerdhem P, Malm J, Ridderstråle M, Akesson K (2014). Vitamin D insufficiency over 5 years is associated with increased fracture risk-an observational cohort study of elderly women. Osteoporos Int.

[16] Thirunthaiyan MR, Mukherjee K, Prashanth T, Kumar DR (2022). Predicting the anatomical location of neck of femur fractures in osteoporotic geriatric Indian population. Malays Orthop J.

[17] Li PF, Lin ZL, Pang ZH, Zeng YR (2016). Does serum calcium relate to different types of hip fracture? A retrospective study. Chin J Traumatol.

[18] Millrose M, Schmidt W, Krickl J, Ittermann T, Ruether J, Bail HJ, Gesslein M (2023). Influence of malnutrition on outcome after hip fractures in older patients. J Pers Med.

[19] Alijanpour K, Afzal S, Alijanpour A, Barati H, Gholinia H, Tavassoli M (2024). Analysis of serum vitamin D and calcium levels in elderly patients with stable and unstable intertrochanteric fractures: a multi-center prospective study. PLoS One.

[20] Oltra G, Burgos MA, Ivaldi D, Escobar Liquitay CM, Franco JV, Garegnani LI (2026). Screening for osteoporosis with bone densitometry in adults with risk factors for fractures. Cochrane Database Syst Rev.

[21] Ghalenavi E, Mirfeizi Z, Hashemzadeh K, Sahebari M, Joker MH (2024). Diagnostic value of radiographic Singh index compared to dual-energy X-ray absorptiometry scan in diagnosing osteoporosis: a systematic review. Arch Bone Jt Surg.

[22] Guo YM, Cheng JH, Zhang H (2022). Serum periostin level and genetic polymorphisms are associated with vertebral fracture in chinese postmenopausal women. Genes (Basel).

[23] Farrokhi M, Arjaki D, Peykanpour F (2020). Circulating periostin levels increase in association with bone density loss and healing progression during the early phase of hip fracture in Chinese older women. Osteoporos Int.

[24] Nethander M, Vandenput L, Eriksson AL, Windahl S, Funck-Brentano T, Ohlsson C (2019). Evidence of a causal effect of estradiol on fracture risk in men. J Clin Endocrinol Metab.

[25] Sharifi MD, Mohebbi M, Farrokhfar M, Farzaneh R, Disfani HF, Hashemian AM (2018). Analysis of correlation between estradiol and fracture of femur neck. Eur J Transl Myol.

[26] Kantharaj C, Murugharaj S (2026). Association of serum estradiol and testosterone with hip fractures in elderly men. Cureus.

